# Editorial: Women in neurogenesis

**DOI:** 10.3389/fnins.2023.1285728

**Published:** 2023-09-14

**Authors:** Margarita Pérez-Martín, Sumana Chakravarty

**Affiliations:** ^1^Departamento de Biología Celular, Genética y Fisiología, Universidad de Málaga, Málaga, Spain; ^2^Instituto de Investigación Biomédica de Málaga y Plataforma en Nanomedicina–IBIMA Plataforma Bionand, Málaga, Spain; ^3^Indian Institute of Chemical Technology (CSIR), Hyderabad, India

**Keywords:** dietary factors, neural stem cell, neurological diseases, neurogenic niches, stress, vitamin C, therapeutic strategies

Research in neurogenesis has proven to be of vital importance in the field of neuroscience and medicine, lately. This process of generating new neurons in the brain plays a fundamental role in development, learning, memory and brain plasticity. In fact, the final acceptance of the “ADULT NEUROGENESIS” concept (1990s) took a long time since its discovery of existence (1960). Understanding how neurons are regenerated and replaced in the adult brain will expand our understanding of the complexity of the human brain, having a significant impact on health, cognition and quality of life.

In the broad exploration of the field of neurogenesis, women scientists have made a significant mark. Their pioneering research and contributions to the field have expanded our understanding of the process of generating new neurons in the brain. However, we cannot mention them all, but a few are worth mentioning here. Bayer et al. (1982) stands out for her important contribution to hippocampal neurogenesis, showing that new neurons continue to be incorporated during juvenile and adult life. Luskin (1993) did enormous work to understand the cellular and molecular mechanisms that regulate neurogenesis, especially in the subventricular zone. More recently, Drapeau et al. (2003) has had a major impact for her work in support of the hypothesis that neurogenesis is involved in memory processes and alterations in the hippocampus. All these data reinforce the hypothesis that neurogenesis continues into adulthood and is involved in memory processes and cognitive alterations related to aging. With the aim of highlighting research in the field of neurogenesis led and carried out by those who identify themselves as women researchers we have proposed a Research Topic entitled “*Women in neurogenesis*,” which is part of a series of initiatives created to increase the visibility of women in science, especially in neuroscience.

This Research Topic is composed of five articles focused on understanding the biology of the NSCs/NPCs and the process of neurogenesis regulation in major neurogenic areas such as the hippocampus and lateral ventricles, as well as in emerging areas such as the hypothalamus.

The current literature provides solid evidence about the fundamental role of dietary factors in hippocampal plasticity. Malnutrition from the fetal stage and/or throughout life contributes to the acceleration of age-related deterioration, positioning diet as an important factor in the risk, progression, and severity of the mental diseases. Melgar-Locatelli et al. summarize the most recent findings related to the influence of nutrition and diet in the modulation of adult hippocampal neurogenesis (AHN). They discuss the importance of maternal nutrition in the AHN of the offspring as well as the role of the microbiota-gut-brain axis in the nutrition-neurogenesis relationship that could act as a link between nutritional factors and AHN. They conclude that nutritional interventions from early stages and throughout life are a promising perspective to alleviate neurodegenerative diseases by stimulating neurogenesis.

In line with the factors contributing to cognitive impairment and subsequent development of neurodegenerative diseases, stress has been identified as an environmental factor that can produce potent an enduring effect on brain structure and function. The effects of stress on hippocampal neurogenesis are widely known, and it has been described that stress strongly suppresses adult hippocampal neurogenesis. Infantes-López et al. characterize the response of the hypothalamus to stress, a new neurogenic area emerged in the las decades. Although the level of neurogenesis in this area is low under physiological conditions, there is a growing interest in clarifying the process of hypothalamic neurogenesis because of the functional implications of this region. Their results are the first to show that even a short-term environmental stimulus such as acute and intense stress can have neuroplastic, inflammatory, functional and metabolic consequences on the adult hypothalamus.

Salazar et al. report on the role of vitamin C and its sodium-dependent vitamin C transporter 2 (SVCT2) in proliferation, differentiation and neurogenesis in embryonic and adult brains. In their review they highlight that vitamin C stimulates neuronal differentiation in all neurogenic niches studied. In addition, they address a new role attributed to vitamin C as a potent epigenetic regulator in stem cells, promoting pluripotency or differentiation and synaptic maturation.

The review by Paez-Gonzalez et al. highlights the role of the ependymal barrier in the context of neurogenesis and homeostasis and discussed future research lines for development of actual therapeutic strategies addressed to the recovery of the ependyma damage after neuroinflammation, especially neuroinflammation triggered by intraventricular hemorrhages in the germinal matrix. They propose that combinatory effects of different stem cell types could be a reasonable therapeutic approach to recover the ependymal function and, therefore, neurogenesis while also reducing the inflammatory response.

Finally, compelling evidence from the last decade suggests that NSCs of the adult SVZ (subventricular zone) may be the cell of origin that encloses the driver mutations of glioblastoma (GBM), the most malignant primary brain tumor in humans. A better understanding of the biology of SVZ NSCs/NPCs will allow a deeper understanding of the etiology of this devastating cancer as well as the search for alternative therapies aimed at attacking the population of tumor-initiating cells. In this sense, the work of Jiménez-Madrona et al. is addressed to analyze whether hemichannel-mediated communication affects proliferation of SVZ NPCs and GBM cells. Their results provide evidence of an antimitotic action of boldine in SVZ NPCs and in GBM cells which may be due, at least in part, to its hemichannel blocking function.

In summary, we believe that the contributions gathered in this Research Topic provide an excellent overview of the state of the art of neurogenesis research, highlighting current promises and limitations and pointing the way forward. Understanding how new neurons are formed and how they are maintained throughout life will lead to a better understanding of the etiology of neurological diseases, allowing the design of therapeutic approaches that consider the stimulation of the neurogenic process.

## Author contributions

MP-M: Writing—original draft, Writing—review and editing. SC: Writing—review and editing.
